# Immersive Virtual Reality for Pain and Anxiety Reduction During Transrectal Ultrasound-Guided Prostate Biopsy: A Systematic Review and Meta-Analysis of Randomized Controlled Trials

**DOI:** 10.3390/jcm15208145

**Published:** 2026-10-20

**Authors:** Khaled Aljenfawi, Malek Hasan, Ebraheem Albazee, Said Yaiesh

**Affiliations:** 1 Kuwait Board of Urology, Kuwait Institute for Medical Specializations (KIMS), Kuwait City 13018, Kuwait; dr.aljenfawi@gmail.com; 2 Royal College of Surgeons in Ireland (RCSI), Busaiteen 15503, Bahrain; malekderbas122@gmail.com; 3 Otorhinolaryngology-Head and Neck Surgery, Kuwait Institute for Medical Specializations (KIMS), Kuwait City 13018, Kuwait; 4 Department of Otolaryngology-Head and Neck Surgery, Al- Jahra Hospital, Al-Jahra 03200, Kuwait; 5 Urology Unit, Department of Surgery, Jaber Al Ahmad Hospital, Kuwait City 91710, Kuwait; syaiesh@hotmail.com

**Keywords:** TRUS, urology, cancer, biopsy, satisfaction

## Abstract

**Background:** Transrectal ultrasound (TRUS) guided prostate biopsy is a distressing procedure associated with significant pain and anxiety, even with routine local anesthesia. Immersive virtual reality (VR) has emerged as a promising non-pharmacological distraction strategy, aiming to modulate pain perception via sensory overload and attentional shift. **Methods:** A comprehensive search of five databases, including PubMed, Web of Science, CENTRAL, Scopus, and Embase, was conducted up to April 2026. RCTs comparing intra-procedural immersive VR to standard care were included. Primary outcomes were biopsy-related pain and anxiety. Secondary outcomes included patient satisfaction and procedure-related adverse events. Standardized mean differences (SMD) were pooled using a random-effects model. **Results:** Four RCTs involving 454 patients were included. There was no significant difference between the two groups regarding biopsy-related pain (*N* = 355 patients, SMD: −1.24, 95% CI [−2.53, 0.04]; *p* = 0.06) and biopsy-related anxiety (*N* = 361 patients, SMD: −1.37, 95% CI [−2.76, 0.02]; *p* = 0.054). These analyses demonstrated significant inter-study heterogeneity (I^2^ > 95%), and leave-one-out sensitivity analyses revealed instability in the pooled estimates. Nevertheless, VR appeared feasible and well tolerated, with no major device-related adverse events reported in the available trials. **Conclusions:** The available evidence remains very uncertain regarding the effect of immersive VR on pain and anxiety during TRUS-guided prostate biopsy. Substantial clinical and statistical heterogeneity and instability of the pooled estimates limit definitive interpretation, and the current evidence is insufficient to support routine implementation. Larger, adequately powered, and standardized RCTs are warranted.

## 1. Introduction

Transrectal ultrasound-guided prostate biopsy (TRUS) remains widely performed for the diagnosis of suspected prostate cancer [1]. However, this procedure is often associated with considerable discomfort for patients [2]. Even with routine administration of local anesthesia, clinically significant discomfort is increasingly reported [3]. This anxiety is significant, with serious consequences including suboptimal patient experience, more procedural complexity, and a reticence to re-biopsy [4,5].

Pain management currently relies on local anesthesia, conscious sedation, and various pharmaceutical modalities [3]. However, these choices carry serious limitations, either providing inadequate control of pain and anxiety or presenting risks of systemic toxicity, logistical challenges, and increased procedure expenses [6,7]. To overcome these pharmacologic obstacles, non-pharmacologic distraction methods were investigated, including music and basic video therapy [8,9]. However, evidence regarding the efficacy of conventional distraction methods such as music and basic video therapy is mixed, with variable effects reported across studies and generally limited certainty regarding the magnitude of benefit. Therefore, there is increasing interest in virtual reality (VR) as a promising, more advanced modality.

The potential effects of VR on pain and anxiety are generally attributed to attentional distraction and immersion. Because attentional resources are limited, engagement with an immersive visual and auditory environment may redirect attention away from procedure-related sensory and emotional stimuli, thereby reducing the perceived intensity or unpleasantness of pain [10]. VR has several benefits, including its non-invasiveness and few adverse effects. VR has shown potential benefits for pain and anxiety reduction in several medical and minor procedural settings, although the magnitude and consistency of these effects vary across populations and procedures [11,12,13]. VR has also been evaluated in office-based urologic procedures. Randomized studies of cystoscopy and vasectomy have examined VR as a distraction strategy for procedural pain and anxiety, with some reporting reductions in anxiety, improved satisfaction, or lower pain in selected analyses [14,15,16]. These findings may support continued investigation of VR as a non-pharmacological adjunct across awake urologic procedures.

Recently, emerging randomized controlled studies (RCTs) have studied the use of VR during TRUS biopsies [17,18,19,20]. However, their results remain inconsistent due to small sample sizes and methodological heterogeneity. Also, the current literature lacks a high-quality synthesis that focuses exclusively on immersive VR during TRUS. Therefore, this systematic review and meta-analysis aims to evaluate the efficacy of immersive VR for pain and anxiety reduction during TRUS-guided prostate biopsy. We hypothesized that immersive VR would be associated with lower biopsy-related pain and anxiety compared with standard care.

## 2. Methods and Materials

### 2.1. Protocol Registration

This systematic review and meta-analysis were prospectively registered in the International Prospective Register of Systematic Reviews (PROSPERO) on [14 August 2026] (ID: CRD420261478646). No amendments or deviations from the registered protocol were made. The study protocol and final manuscript were prepared in accordance with the Preferred Reporting Items for Systematic Reviews and Meta-Analyses (PRISMA) statement (Supplemental PRISMA Checklist, Table S1) [21].

### 2.2. Data Sources & Search Strategy

A comprehensive electronic literature search was conducted up to 26 April 2026 across PubMed, Web of Science, Cochrane Central Register of Controlled Trials (CENTRAL), Scopus, and Embase. The search strategy utilized the following search string: (“virtual reality” OR “smart glass*” OR “immersive” OR “non-immersive” OR “head-mounted display” OR “augmented reality” OR “mixed reality” OR “virtual therapy” OR “virtual environment” OR “virtual treatment” OR “visual distract*” OR “audiovisual distract*” OR “photic stimulation” OR “motion picture*” OR “watch* video*”) AND (“Prostate Biopsy” OR TRUS OR “transrectal prostate biopsy” OR “transrectal ultrasound guided prostate biopsy” OR “TRUS-PB”). Also, the reference lists of eligible trials were manually cross-checked to identify any additional relevant studies. The detailed search strings for each database are provided in Table S2.

### 2.3. Eligibility Criteria

We included RCTs evaluating VR distraction-based interventions according to the following PICO framework:

- **Population (P):** Adult patients undergoing transrectal prostate biopsy, regardless of indication.
- **Intervention (I):** immersive VR distraction-based interventions applied during the biopsy procedure. Immersive VR was defined as visual content delivered through an enclosed head-mounted VR device or headset that replaced the participant’s external visual field with a virtual environment. Interactivity was not required for eligibility and was considered a separate intervention characteristic; therefore, a passive 360° video delivered through a VR headset was considered immersive.
- **Comparison (C):** Standard care, no intervention, or alternative non-distraction control conditions.
- **Outcomes (O):** The primary outcomes were pain perception and anxiety. Secondary outcomes included patient comfort/satisfaction, physiological parameters (heart rate, blood pressure), procedural metrics, and complication rates.

Only full-text reports were eligible for inclusion; conference abstracts without an available full-text report were excluded. Studies that had been accepted for publication or were available in press were eligible, provided that a complete full-text report containing sufficient methodological and outcome data was available. No language restrictions were applied. Additionally, we excluded: non-randomized studies (observational, retrospective, case series), studies evaluating audio-only interventions without a visual component, trials conducted in procedures other than prostate biopsy, and studies evaluating non-immersive audiovisual distraction (video glasses, audiovisual media).

### 2.4. Study Selection

Retrieved records were imported into Covidence software for automated de-duplication. Two independent reviewers (KA & MH) screened the remaining articles by title and abstract, followed by a rigorous full-text appraisal of those that met the initial screening criteria. Any discrepancies regarding study eligibility were resolved through consensus or consultation with a senior author (EA). Covidence’s automated non-RCT exclusion feature was used as an initial screening aid to identify and exclude records classified as non-randomized studies, consistent with our prespecified eligibility criterion restricting inclusion to RCTs. The automation was applied only to study design classification; all remaining eligibility criteria were assessed independently by the reviewers during title/abstract and full-text screening.

### 2.5. Data Extraction

Data were independently extracted by two reviewers (KA & MH) into a standardized Microsoft Excel spreadsheet. The extracted variables covered three main domains: (1) Study characteristics (study ID, country, study design, visual content, VR device, distraction timing, control, TRUS procedure details, pain assessment tool, anxiety assessment tool, sedation, main inclusion criteria, and primary outcome); (2) Baseline patient characteristics (number of patients in each group, age, previous biopsy, baseline pain, baseline anxiety, local anesthesia); and (3) Outcome data as defined by our PICO criteria. Disagreements during extraction were resolved through consensus.

### 2.6. Risk of Bias and Certainty of Evidence

The risk of bias for each included RCT was independently assessed by at least two reviewers (KA & MH) using the Cochrane Risk of Bias 2 (RoB-2) tool [22]. This tool evaluates bias across five domains: randomization process, deviations from intended interventions, missing outcome data, measurement of the outcome, and selection of the reported result. The overall certainty of the synthesized evidence was assessed using the Grading of Recommendations Assessment, Development, and Evaluation (GRADE) framework [23,24]. This framework downgrades evidence based on identified limitations such as risk of bias, inconsistency, indirectness, and imprecision. All judgments were documented, and any disagreements were resolved by consensus.

### 2.7. Statistical Analysis

All statistical analyses were conducted using Stata, version 19 (StataCorp). Continuous outcomes were pooled using standardized mean differences (SMDs) with corresponding 95% confidence intervals (CIs), because the included studies assessed the same underlying constructs (pain, anxiety, and satisfaction) using different measurement scales. A restricted maximum likelihood (REML) random-effects model was used in the primary analysis to account for clinical and methodological heterogeneity across the included studies. Study estimates were pooled using inverse-variance weighting, with between-study variance estimated by REML, and 95% CIs for the pooled effects were calculated using the normal approximation. Because only two or three studies contributed to each pooled outcome, estimates of the pooled effect and between-study variance may be sensitive to individual studies, and the resulting CIs should therefore be interpreted cautiously. Inter-study heterogeneity was assessed via Cochran’s Q (Chi-squared test) and the I^2^ statistic, with significant heterogeneity defined as *p* < 0.10 or I^2^ > 50%, respectively. Finally, assessment of publication bias via funnel plots and Egger’s test was not performed because the meta-analysis included fewer than 10 studies per outcome, rendering these tests underpowered [25].

## 3. Results

### 3.1. Search Results and Study Selection

**Figure 1:**
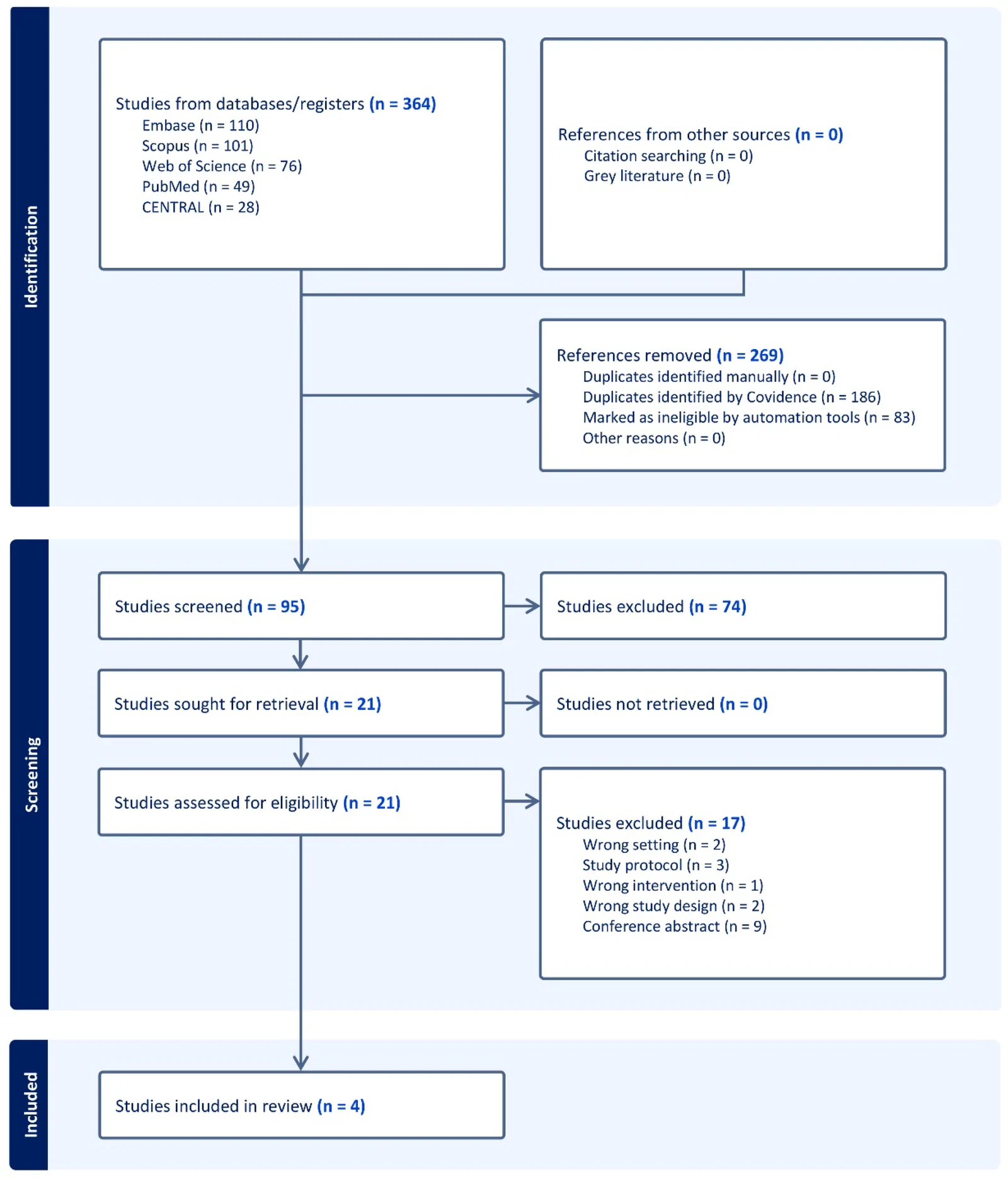
PRISMA flow diagram of study selection. The diagram illustrates the process of literature identification, screening, eligibility assessment, and study inclusion in the systematic review.

A total of 364 records were identified from the initial literature search across five databases. After removing 269 records (186 duplicates identified by Covidence and 83 marked as ineligible by automation tools), 95 articles remained for title and abstract screening. Of these, 74 records were excluded. Twenty-one full-text articles were sought and successfully retrieved. Seventeen studies were excluded for various reasons (Table S3). Finally, four trials [17,18,19,20] were included in the review (Figure 1).

### 3.2. Characteristics of Included Studies

Four RCTs comprising 454 patients were included in this analysis [17,18,19,20]. Across trials, baseline characteristics were comparable between the intervention and control groups, although inclusion criteria and exclusion criteria (prior biopsy, psychiatric illness, or sensory impairment) varied slightly across studies. Another source of variability was the type of intervention; while all studies evaluated VR as a distraction technique during biopsy, the implementation differed. Bozkul et al. assessed anxiety but not pain [17], whereas Genç et al. assessed pain but not anxiety [18]. Accordingly, three RCTs contributed to the pain meta-analysis [18,19,20], and three RCTs contributed to the anxiety meta-analysis [17,19,20]. Detailed study designs and baseline patient characteristics are summarized in the main tables (Table 1 and Table 2).

### 3.3. Risk of Bias and Certainty of Evidence

**Figure 2:**
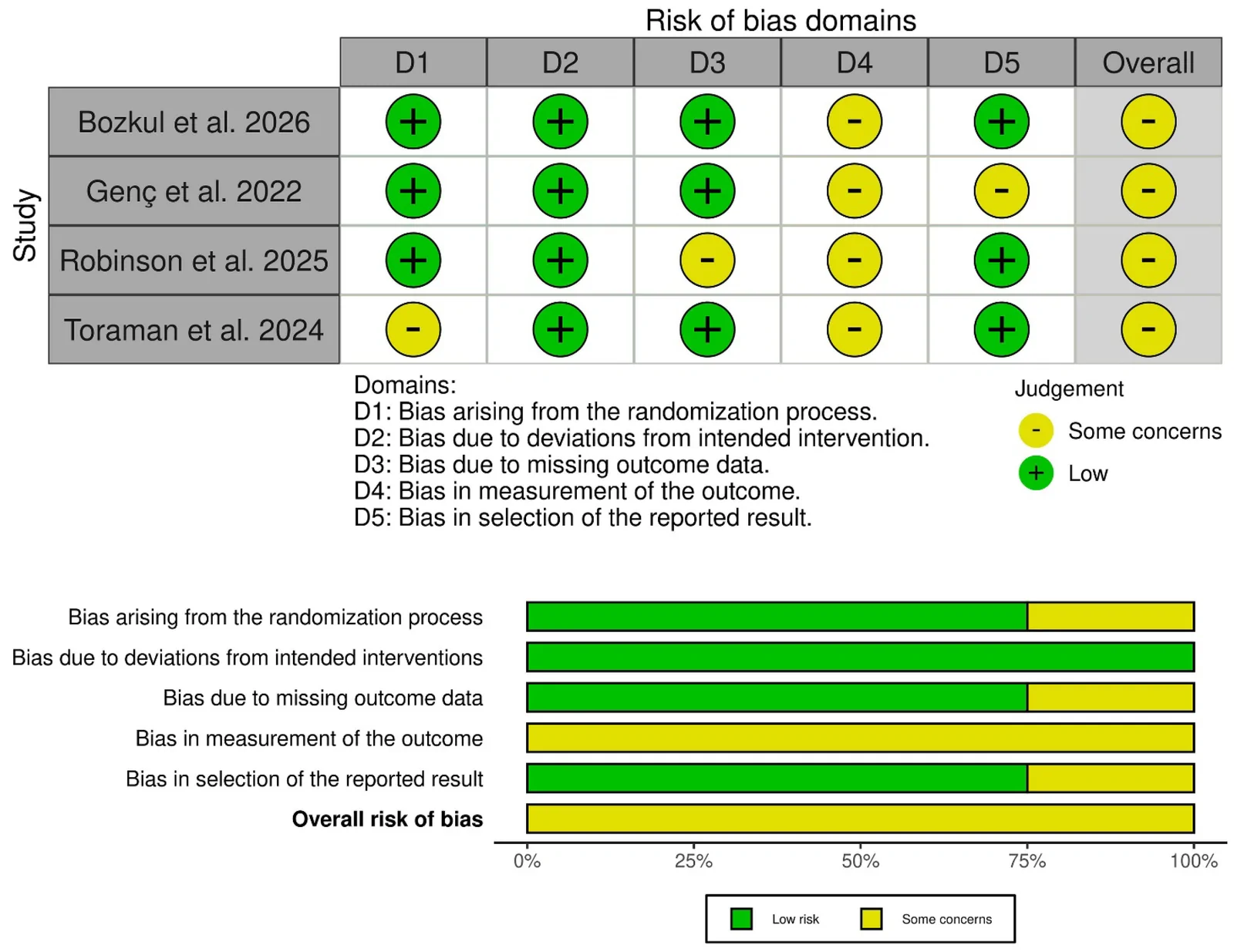
Risk-of-bias assessment of the included randomized controlled trials using the RoB 2 tool. The upper panel summarizes domain-level judgments for each study (D1–D5). In contrast, the lower panel shows the overall proportion of studies rated as low risk or with some concerns for each bias domain [17,18,19,20].

Figure 2 presents the risk-of-bias assessment of the included RCTs using the RoB-2 tool. All the studies included were judged to have some concerns regarding the overall risk of bias. This was primarily driven by consistent concerns about bias in the measurement of the outcome, reflecting the subjective nature of outcomes such as pain and anxiety, and the lack of blinding. Detailed RoB 2 signaling-question responses and study-specific supporting justifications are provided in Table S4. Additionally, the certainty of evidence assessment details are highlighted in Table 3.

## 4. Primary Outcomes

### 4.1. Biopsy-Related Pain

**Figure 3:**
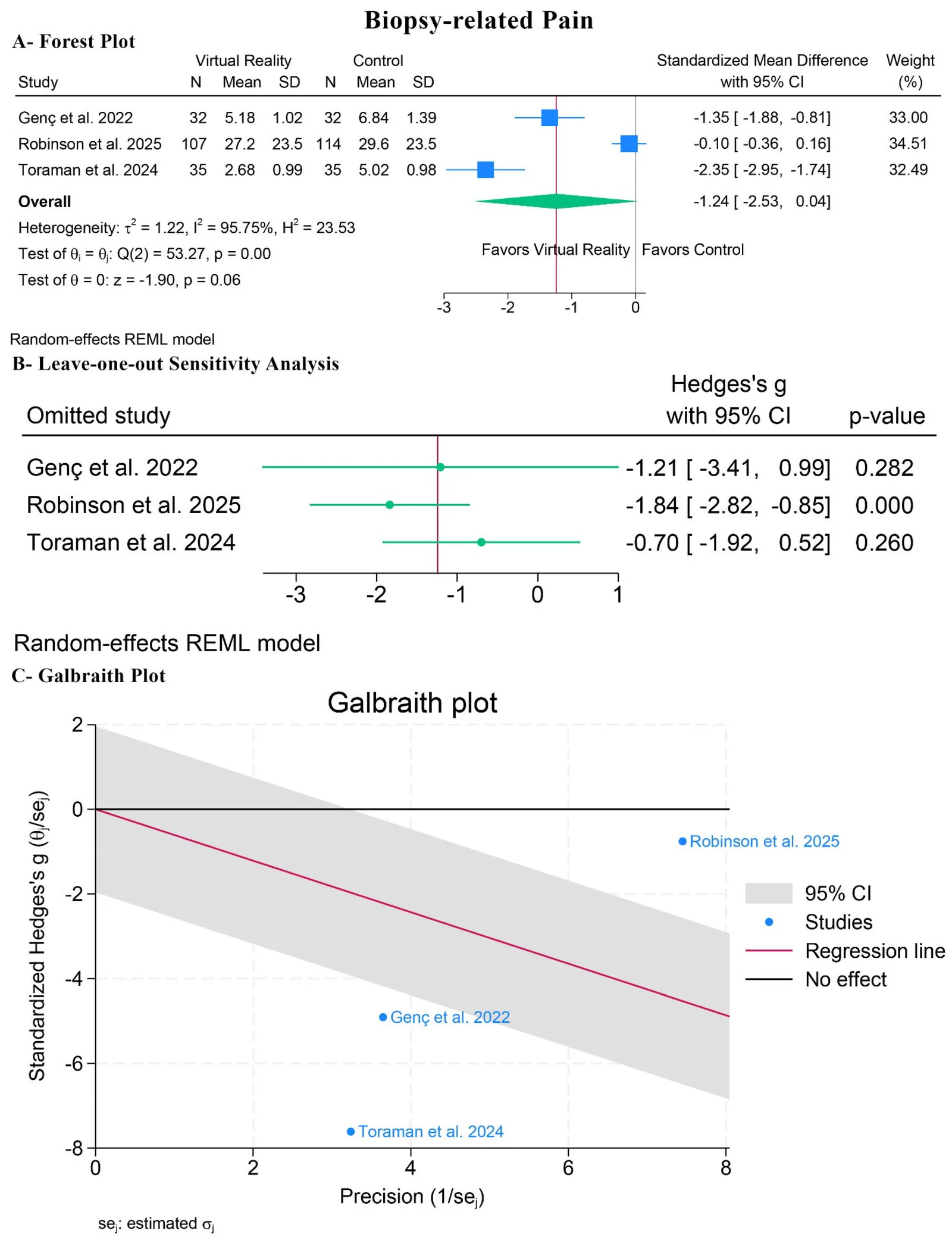
Biopsy-related pain. (**A**) Forest plot showing the standardized mean difference (SMD) in biopsy-related pain between virtual reality and control groups using a random-effects model. (**B**) Leave-one-out sensitivity analysis demonstrating the influence of each study on the pooled effect estimate. (**C**) Galbraith plot assessing heterogeneity and identifying potential outlier studies [18,19,20].

Three RCTs (Genç et al., Robinson et al., and Toraman et al.) contributed to the pain meta-analysis. There was no significant difference between the two groups (SMD: −1.24, 95% CI [−2.53, 0.04], *p* = 0.06, I^2^ = 95.75%, τ^2^ = 1.22) (Figure 3A). Leave-one-out sensitivity analysis demonstrated sensitivity to individual studies. The pooled SMD ranged from −0.70 (95% CI [−1.92, 0.52]) when Toraman et al. was omitted to −1.84 (95% CI [−2.82, −0.85]) when Robinson et al. was omitted; the omission of Genç et al. yielded an SMD of −1.21 (95% CI [−3.41, 0.99]). These changes in effect magnitude and precision indicate instability of the pooled estimate (Figure 3B). The Galbraith plot showed notable dispersion of studies around the regression line, indicating substantial heterogeneity (Figure 3C). The timing of pain assessment varied slightly across studies. Genç et al. assessed pain severity experienced during the biopsy using a 0–10 VAS, with patients asked to rate this 15 min after completion of the procedure [18]. Robinson et al. assessed “worst pain during procedure” using a 0–100 VAS-based item administered immediately after the procedure [19]. Toraman et al. used a 0–10 VAS for pain intensity administered 10 min after completion of the biopsy [20].

### 4.2. Biopsy-Related Anxiety

**Figure 4:**
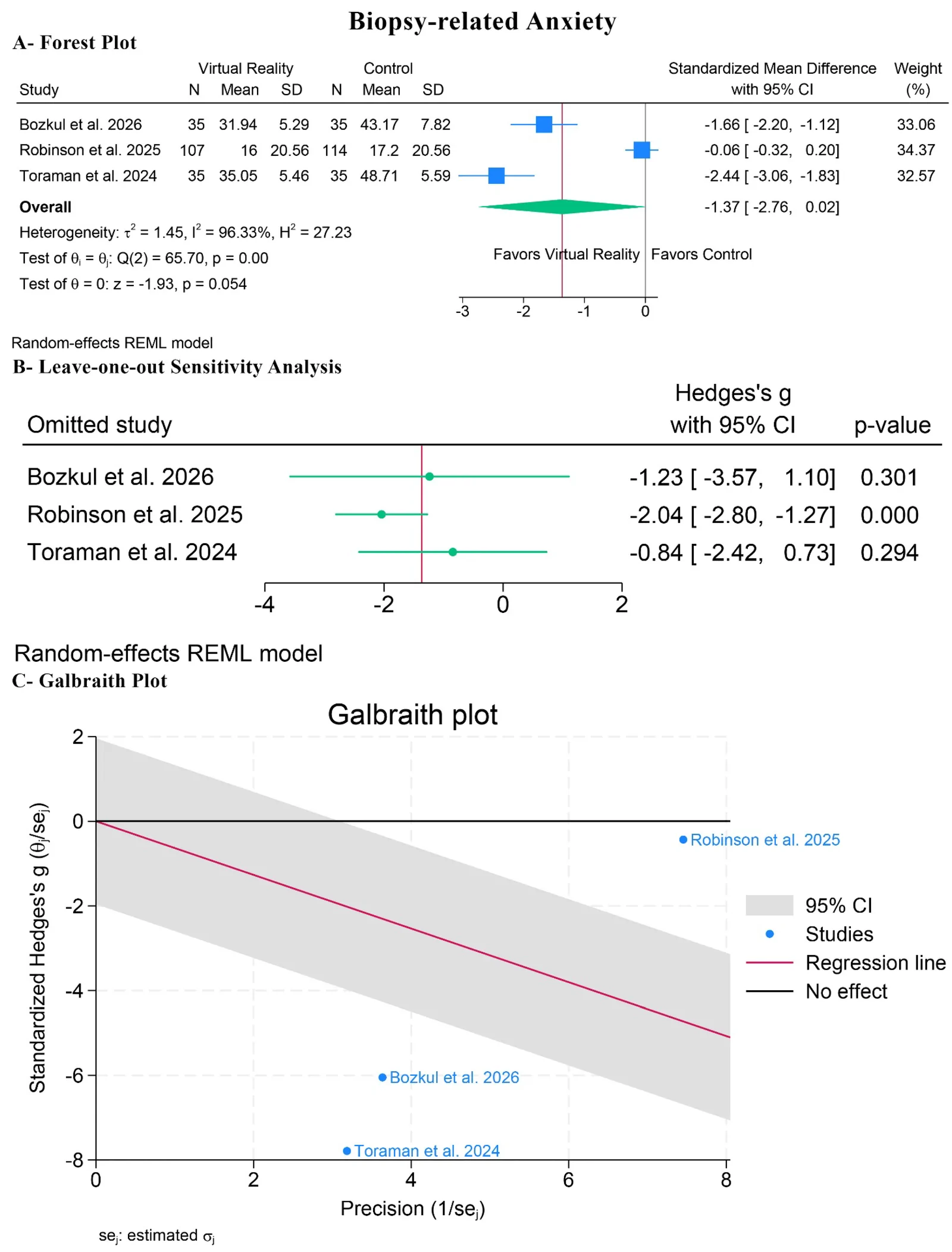
Biopsy-related anxiety. (**A**) Forest plot showing the standardized mean difference (SMD) in biopsy-related anxiety between virtual reality and control groups using a random-effects model. (**B**) Leave-one-out sensitivity analysis demonstrating the influence of each study on the pooled effect estimate. (**C**) Galbraith plot illustrating between-study heterogeneity and the distribution of individual study effects [17,19,20].

Three RCTs (Bozkul et al., Robinson et al., and Toraman et al.) contributed to the anxiety meta-analysis. There was no significant difference between the two groups (SMD: −1.37, 95% CI [−2.76, 0.02], *p* = 0.054, I^2^ = 96.33%, τ^2^ = 1.45) (Figure 4A). Leave-one-out sensitivity analysis demonstrated sensitivity to individual studies. The pooled SMD ranged from −0.84 (95% CI [−2.42, 0.73]) when Toraman et al. was omitted to −2.04 (95% CI [−2.80, −1.27]) when Robinson et al. was omitted; with the omission of Bozkul et al. yielding an SMD of −1.23 (95% CI [−3.57, 1.10]) (Figure 4B). The Galbraith plot showed notable dispersion of studies around the regression line, indicating substantial heterogeneity (Figure 4C). Anxiety was assessed immediately after the procedure in Bozkul et al. [17] and Robinson et al. [19], whereas Toraman et al. [20] performed the post-procedural assessment 10 min after biopsy completion.

## 5. Secondary Outcomes

### 5.1. Patient Satisfaction

Patient satisfaction was reported in two trials using different measures and was therefore not quantitatively pooled. Toraman et al. assessed patients’ perception of nursing care using the 15-item PPHEN scale and reported a higher post-procedure score in the VR group than in the control group (65.42 ± 4.65 vs. 42.40 ± 4.52; *p* < 0.001) [20]. In contrast, Robinson et al. assessed overall satisfaction with the biopsy procedure and found similar scores between the VR and control groups (79.2 vs. 77.1; MD: 2.1, 95% CI [−4.4, 8.7]; *p* = 0.520) [19].

### 5.2. Physiological Parameters (Vital Signs)

Detailed physiological outcomes were only reported by Genç et al. VR was associated with significant improvements in vital signs, including reductions in diastolic blood pressure, pulse rate, and respiratory rate, along with an increase in oxygen saturation (*p* < 0.05) [18].

### 5.3. Procedural Experience and Tolerability

In Robinson et al., VR was not associated with a statistically significant difference in procedural experience measures, including time thinking about pain (MD: 0.9, 95% CI [−7.2, 9.0]) and perceived unpleasantness (MD: −3.3, 95% CI [−10.0, 3.4]) [14]. Similarly, willingness to undergo repeat biopsy was comparable between groups. The results indicate that, despite advancements in user experience, VR did not consistently improve measures of procedural experience or tolerability.

### 5.4. Complications & Adverse Events

Safety and adverse-event reporting were limited and heterogeneous across the included trials. Bozkul et al. systematically assessed biopsy-related complications in all 70 participants during the first 24 h after the procedure. Urinary retention occurred in 0/35 patients in the VR group and 5/35 in the control group, whereas no clear between-group differences were observed for the other recorded complications [17]. Robinson et al. assessed several procedural and post-procedural symptoms; intra-procedural nausea scores were lower with VR (MD: −4.2, 95% CI [−8.3, −0.1]), whereas post-procedural nausea and vomiting were similar between groups (MD: 2.6, 95% CI [−1.2, 6.4]) [19]. These secondary findings should be considered exploratory. No major VR-related adverse events were reported in the available trials; however, adverse events were not systematically assessed across all studies, and the available evidence should therefore not be interpreted as establishing the safety of VR.

### 5.5. Feasibility and Acceptability

Available reports suggest that VR was generally feasible and well tolerated during biopsy. Bozkul et al. reported no VR-related interruption or device removal among the 35 patients receiving VR [17], and Robinson et al. reported no VR-related harms [19]. However, systematic assessment of VR-specific symptoms and device-related discontinuation was not undertaken consistently across all trials.

## 6. Discussion

Our synthesis of four trials and 454 patients showed that intra-procedural immersive VR had a statistically non-significant trend toward reducing biopsy-related pain and anxiety. Leave-one-out analyses demonstrated that the pooled estimates were sensitive to the exclusion of Robinson et al. [19], which resulted in statistically significant pooled effects for both pain and anxiety. This finding should not be interpreted as evidence that the Robinson trial distorted the overall result. Still, it highlights the instability of pooled estimates derived from a small and heterogeneous evidence base. Beyond these primary endpoints, the two trials assessing patient satisfaction used different constructs and produced differing findings, precluding a meaningful pooled synthesis. Finally, VR was generally well tolerated, with no major device-related adverse events reported.

Methodological differences across included trials might explain the significant statistical heterogeneity. First, VR content and devices varied: Robinson et al. [19] utilized an interactive Oculus Go headset, whereas Genç et al. [18], Bozkul et al. [17], and Toraman et al. [20] used passive nature videos via smartphone-based headsets. Second, exposure timing differed; Toraman et al. [20] initiated VR pre-procedure to target baseline anxiety, while others restricted it to the 10-min intra-procedural window [17,18,19]. Third, differences in background analgesia and anesthesia protocols may have contributed to the observed heterogeneity. VR may exert a larger relative effect when background analgesia is limited, whereas concomitant local anesthesia may reduce the additional benefit achievable through distraction; however, this remains a hypothesis and cannot be established from the small number of available trials. Fourth, studies used different scales to assess pain and anxiety, with outcomes measured at different post-procedural time points. Finally, the populations differed in age, geographic setting, previous biopsy experience, and familiarity with VR technology.

One proposed explanation for the potential effects of VR on pain and anxiety is attentional distraction [17]. Immersive visual and auditory stimuli may redirect attention away from procedure-related sensory and emotional inputs, which could reduce perceived pain or distress. Engagement with visual and auditory stimuli may redirect attention away from procedure-related sensory and emotional stress, potentially modifying the perceived intensity of pain [26,27]. The physiological changes reported by Genç et al. [18], including lower heart rate and diastolic blood pressure, are compatible with a reduction in physiological stress but do not establish attentional distraction or immersion as the causal mechanism.

Additionally, compared to traditional passive distractions such as music, immersive VR needs greater cognitive engagement by creating a psychological illusion of physical presence in the virtual environment [28,29]. However, the success of this mechanism is highly variable, as it depends on the hardware’s level of immersion, the software’s interactivity, and the individual patient’s technological engagement [30].

Moreover, our inconclusive findings differ from some reports in other procedural settings, where immersive VR has been associated with reductions in pain or distress. However, effects across the broader literature are heterogeneous and may depend on the procedure, population, VR content, and implementation. Meta-analyses in burn and wound-care settings have reported reductions in procedural pain with VR in some contexts, yielding benefits during repetitive debridement [31,32]. Similarly, studies in pediatric procedures and awake endoscopic settings have reported reductions in procedural distress or pain, although findings are not uniformly consistent across studies [12,33]. Differences between the present findings and those reported in other clinical settings may relate to distinct procedural characteristics of the TRUS procedure. Unlike burn care, which involves sustained superficial somatic pain, a prostate biopsy entails acute, rapid, and repetitive nociceptive stimulation associated with prostate sampling [34,35].

Additionally, the baseline anxiety associated with the possibility of a cancer diagnosis imposes a significant psychological strain that superficial VR distraction may not be able to overcome [36]. Also, the modern standard of care requires the routine use of highly efficacious local anesthesia [37]. This can limit the benefit VR can provide, preventing the technology from demonstrating the larger treatment effects observed in procedures without targeted nerve blocks.

Despite the inherent limitations of the literature, this meta-analysis, to the best of our knowledge, is the first comprehensive synthesis to focus exclusively on immersive VR during TRUS-guided prostate biopsy. Still, it is necessary to acknowledge several inherent limitations. First, the meta-analysis is limited by the small number of eligible studies (*n* = 4), which limits the precision of heterogeneity estimates and contributes to instability in the pooled and leave-one-out analyses. Second, the trials lacked standardization, with varying VR hardware, exposure durations, and psychometric scales. Third, using unblinded subjective endpoints may introduce detection bias, and the low trial count prevented us from formal mathematical assessments of publication bias.

Fourth, generalizability is limited by the focus on the transrectal approach, given the increasing use of transperineal biopsy. Emerging evidence suggests that VR may also be feasible in this setting. In a prospective trial of 60 men undergoing transperineal biopsy under local anesthesia, immersive VR was associated with lower peri-procedural pain and anxiety than standard care [38]. More research using VR-based hypnosis has also suggested good tolerability during prostate biopsy, including transperineal procedures [39]. However, the available evidence remains limited and is not yet sufficient for firm conclusions. Also, the included trials mostly included patients undergoing their first biopsy; the utility of VR in the highly distressed, repeat-biopsy population remains unexamined, and the geographic concentration of the trials (three of four conducted in Turkey) may limit broad cultural applicability. Finally, the overall certainty of the synthesized evidence, evaluated via the GRADE framework, was classified as very low, warranting careful interpretation.

Clinically, immersive VR appeared to be a well-tolerated, feasible, and technologically accessible intervention that does not interfere with urological procedures. The available evidence suggests that VR can be implemented without major disruption to the biopsy procedure and appears generally well tolerated. However, its clinical efficacy remains uncertain, and the current evidence is insufficient to support routine use or to identify specific patient subgroups most likely to benefit. It should not replace optimized local anesthesia methods, such as the periprostatic nerve block.

Future studies should focus on multi-center RCTs, ensuring consistent VR equipment, uniform interactive material, and standardized measurement tools to overcome the inconsistencies that limited this review. Also, direct multi-arm comparisons evaluating VR against music therapy, standard care, and systemic sedation can be very helpful, and further investigation into VR’s efficacy for transperineal biopsies is recommended. Finally, health economics research should also be conducted to evaluate the cost-effectiveness of integrating VR into clinical practice, accounting for patient acceptance over time and the efficiency of patient tolerability.

## 7. Conclusions

The available evidence is very uncertain regarding the effect of immersive VR on pain and anxiety during TRUS-guided prostate biopsy. The small number of trials, substantial clinical and statistical heterogeneity, and instability of the pooled estimates preclude definitive conclusions regarding its efficacy. Current evidence is therefore insufficient to support routine implementation of VR in this setting, and larger, adequately powered, standardized RCTs are needed.

## Figures and Tables

**Table 1:** Summary characteristics of the included RCTs.

Study ID	Country	Study Design	Sample	Visual Content	VR Device	Distraction Timing	Control	TRUS Procedure Details	Pain Assessment Tool	Anxiety Assessment Tool	Sedation	Main Inclusion Criteria	Primary Outcome
Bozkul et al. 2026 [17]	Turkey	RCT	*N* = 70	Six-minute 360° nature-based visual content looped with calming music.	VR Shinecon G12 3D headset with a Xiaomi Redmi Note 8 Pro phone.	Continuously throughout the biopsy (a 30-min VR session).	Standard clinical care, verbal information, and routine nurse-patient interaction.	Left lateral decubitus positioning, periprostatic nerve blockade, and standardized 12-core sampling with an 18-gauge Tru-Cut needle.	Not assessed	State Anxiety Inventory (SAI)	Local anesthetic (bupivacaine); patients using anxiolytics were excluded.	>18 years, oriented/cooperative, first-time VRG user, no anxiety or psychiatric disorders, and no anxiolytic use.	Anxiety and comfort.
Genç et al. 2022 [18]	Turkey	RCT	*N* = 64	10 min of 360° nature video scenes recorded on YouTube.	A smart mobile phone connected to VR glasses.	10 min during the biopsy procedure.	Routine procedure without any additional interventions.	Left lateral position on a stretcher with both legs pulled towards the abdomen.	VAS-10	Not assessed	No anesthetic agent was used; analgesic users were excluded.	>18 years, no previous biopsy, no psychiatric diseases, no severe vision/hearing problems, and no analgesics ≤ 24 h prior.	Pain and vital signs.
Robinson et al. 2025 [19]	New Zealand	RCT	*N* = 250	Pre-selected interactive virtual experience.	Oculus Go VR headset.	Throughout the procedure.	Standard TRUS Bx care without any additional intervention.	The outpatient setting procedure was performed following standard hospital protocols.	VAS-100	VAS-based scale for anxiety	Local anesthetic.	Men undergoing their first TRUS Bx; excluded those with vision/cognitive impairment or active infections.	Procedural pain and anxiety.
Toraman et al. 2024 [20]	Turkey	RCT	*N* = 70	360° videos from YouTube with background music depicting parks, nature, seascapes, underwater scenes, or museums.	VR glasses connected to a smartphone.	From 2 min before the physician’s arrival until the procedure’s end (approx. 10–15 min).	Routine nursing care.	Patients were placed in the left lateral position and underwent the procedure by the same surgeon.	VAS-10	State-Trait Anxiety Inventory (STAI-I)	Excluded if using painkillers, sedatives, anti-anxiety, or psychotropic medications.	18–75 years old, undergoing first TRUS-Bx, and devoid of mental, hearing, vision, or communication problems.	Pain, anxiety, and patient satisfaction.

RCT, randomized controlled trial; VR, virtual reality; TRUS, transrectal ultrasound; Bx, biopsy; VAS, visual analog scale; SAI, State Anxiety Inventory; STAI, State-Trait Anxiety Inventory.

**Table 2:** Baseline characteristics of the participants.

Study ID	Group	Number of Patients in Each Group	Age (Years), Mean (SD)	Previous Biopsy, *N* (%)	Baseline Pain, Mean (SD)	Baseline Anxiety, Mean (SD)	Local Anesthesia, *N* (%)
Bozkul et al. 2026 [17]	VR	*N* = 35	68.62 (7.32)	22 (62.9%)	NR	41.80 (8.47)	35 (100%)
	Control	*N* = 35	69.82 (8.32)	22 (62.9%)	NR	43.80 (7.80)	35 (100%)
Genç et al. 2022 [18]	VR	*N* = 32	70.59 (7.92)	0	0.03 (0.17)	NR	0
	Control	*N* = 32	73.12 (8.21)	0	0.06 (0.24)	NR	0
Robinson et al. 2025 [19]	VR	*N* = 125	64.1 (6.7)	0	NR	36.9 (28.1)	125 (100%)
	Control	*N* = 125	64.6 (7.5)	0	NR	38.0 (28.2)	125 (100%)
Toraman et al. 2024 [20]	VR	*N* = 35	64.11 (5.34)	0	1.22 (0.84)	50.54 (9.95)	0
	Control	*N* = 35	64.31 (4.52)	0	1.20 (1.23)	49.94 (8.48)	0

VR, virtual reality; SD, standard deviation; *N*, number; %, percentage; NR, not reported.

**Table 3:** GRADE evidence profile.

Participants (Studies)	Risk of Bias	Inconsistency	Indirectness	Imprecision	Publication Bias	Overall Certainty of Evidence	Effect Estimates
**Biopsy-related Pain**							
355 (3 RCTs)	serious ^a^	very serious ^b^	not serious	serious ^c^	not assessed	⊕◯◯◯ Very low ^a,b,c^	SMD 1.24 SD lower (2.53 lower to 0.04 higher)
**Biopsy-related Anxiety**							
361 (3 RCTs)	serious ^a^	very serious ^b^	not serious	serious ^c^	not assessed	⊕◯◯◯ Very low ^a,b,c^	SMD 1.37 SD lower (2.76 lower to 0.02 higher)

CI: confidence interval; RCT: randomized controlled trial; SMD: standardized mean difference; Explanations: ^a^. Most trials showed some concerns of bias, mainly due to subjective outcome measurement and lack of blinding. ^b^. Very serious heterogeneity (I^2^ > 95%). ^c^. Wide confidence interval from little or no effect to a potentially large benefit, together with a small number of contributing studies and participants.

## Data Availability

All data are available within the manuscript and can be obtained from the corresponding author upon a reasonable request.

## Supplementary Materials

The following supporting information can be downloaded at: https://www.mdpi.com/article/10.3390/jcm15208145/s1, Table S1. PRISMA 2020 checklist. Table S2: Search strategy. Table S3: Excluded records in full-text screening. Table S4: Detailed RoB 2 signaling-question responses and study-specific supporting justifications.
