# Supplementary material for: Immersive Virtual Reality for Pain and Anxiety Reduction During Transrectal Ultrasound-Guided Prostate Biopsy: A Systematic Review and Meta-Analysis of Randomized Controlled Trials
Source: J Clin Med. 2026 Oct 20;15(20):8145. doi: 10.3390/jcm15208145 (PMC13705641; doi:10.3390/jcm15208145)
Supplement: Supplementary file 1 [file jcm-15-08145-s001.zip › jcm-4598951-supplementary.pdf]

Supplementary Materials:

Contents:

Tables.

Table S1. PRISMA 2020 checklist.
Table S2: Search strategy.
Table S3: Excluded records in full-text screening.
Table S4: Detailed RoB 2 signaling-question responses and study-specific supporting justifications.

Table S1. PRISMA 2020 Checklist

**Section / Topic**	**Item #**	**PRISMA 2020 checklist item**	**Location where item is reported**
TITLE	**1**	Identify the report as a systematic review.	p. 1, Title
ABSTRACT	**2**	See the PRISMA 2020 for Abstracts checklist.	pp. 1–2, Abstract
INTRODUCTION Rationale	**3**	Describe the rationale for the review in the context of existing knowledge.	p. 2, Introduction
INTRODUCTION Objectives	**4**	Provide an explicit statement of the objective(s) or question(s) the review addresses.	p. 2, Introduction
METHODS Eligibility criteria	**5**	Specify the inclusion and exclusion criteria for the review and how studies were grouped for the syntheses.	p. 3, Eligibility Criteria
METHODS Information sources	**6**	Specify all databases, registers, websites, organisations, reference lists and other sources searched or consulted to identify studies. Specify the date when each source was last searched or consulted.	p. 3, Data Sources & Search Strategy
METHODS Search strategy	**7**	Present the full search strategies for all databases, registers and websites, including any filters and limits used.	p. 3, Data Sources & Search Strategy; Supplementary Table S2
METHODS Selection process	**8**	Specify the methods used to decide whether a study met the inclusion criteria of the review, including how many reviewers screened each record and each report retrieved, whether they worked independently, and if applicable, details of automation tools used in the process.	p. 4, Study Selection
METHODS Data collection process	**9**	Specify the methods used to collect data from reports, including how many reviewers collected data from each report, whether they worked independently, any processes for obtaining or confirming data from study investigators, and if applicable, details of automation tools used in the process.	p. 4, Data Extraction
METHODS Data items	**10a**	List and define all outcomes for which data were sought. Specify whether all results that were compatible with each outcome domain in each study were sought (e.g. for all measures, time points, analyses), and if not, the methods used to decide which results to collect.	pp. 3–4, Eligibility Criteria and Data Extraction
METHODS Data items	**10b**	List and define all other variables for which data were sought (e.g. participant and intervention characteristics, funding sources). Describe any assumptions made about any missing or unclear information.	p. 4, Data Extraction
METHODS Study risk of bias assessment	**11**	Specify the methods used to assess risk of bias in the included studies, including details of the tool(s) used, how many reviewers assessed each study and whether they worked independently, and if applicable, details of automation tools used in the process.	p. 4, Risk of Bias and Certainty of Evidence
METHODS Effect measures	**12**	Specify for each outcome the effect measure(s) (e.g. risk ratio, mean difference) used in the synthesis or presentation of results.	p. 4, Statistical Analysis
METHODS Synthesis methods	**13a**	Describe the processes used to decide which studies were eligible for each synthesis (e.g. tabulating the study intervention characteristics and comparing against the planned groups for each synthesis (item #5)).	pp. 3–4, Eligibility Criteria and Data Extraction; p. 6, Characteristics of Included Studies
METHODS Synthesis methods	**13b**	Describe any methods required to prepare the data for presentation or synthesis, such as handling of missing summary statistics, or data conversions.	No additional data conversions or imputations were reported.
METHODS Synthesis methods	**13c**	Describe any methods used to tabulate or visually display results of individual studies and syntheses.	pp. 7–13, Tables 1–3 and Figures 2–4; Supplementary Figure S1
METHODS Synthesis methods	**13d**	Describe any methods used to synthesize results and provide a rationale for the choice(s). If meta-analysis was performed, describe the model(s), method(s) to identify the presence and extent of statistical heterogeneity, and software package(s) used.	pp. 4–5, Statistical Analysis
METHODS Synthesis methods	**13e**	Describe any methods used to explore possible causes of heterogeneity among study results (e.g. subgroup analysis, meta-regression).	pp. 9–12, Figures 3C and 4C; pp. 14–15, Discussion

METHODS Synthesis methods	**13f**	Describe any sensitivity analyses conducted to assess robustness of the synthesized results.	pp. 9–12, Leave-one-out sensitivity analyses (Figures 3B and 4B)
METHODS Reporting bias assessment	**14**	Describe any methods used to assess risk of bias due to missing results in a synthesis (arising from reporting biases).	pp. 4–5, Statistical Analysis (formal assessment not performed because <10 studies per outcome)
METHODS Certainty assessment	**15**	Describe any methods used to assess certainty (or confidence) in the body of evidence for an outcome.	p. 4, Risk of Bias and Certainty of Evidence
RESULTS Study selection	**16a**	Describe the results of the search and selection process, from the number of records identified in the search to the number of studies included in the review, ideally using a flow diagram.	p. 5, Search Results and Study Selection; Figure 1
RESULTS Study selection	**16b**	Cite studies that might appear to meet the inclusion criteria, but which were excluded, and explain why they were excluded.	Supplementary Table S3
RESULTS Study characteristics	**17**	Cite each included study and present its characteristics.	pp. 6–8, Characteristics of Included Studies; Tables 1 and 2
RESULTS Risk of bias in studies	**18**	Present assessments of risk of bias for each included study.	p. 8, Risk of Bias and Certainty of Evidence; Figure 2
RESULTS Results of individual studies	**19**	For all outcomes, present, for each study: (a) summary statistics for each group (where appropriate) and (b) an effect estimate and its precision (e.g. confidence/credible interval), ideally using structured tables or plots.	pp. 9–13, Primary and Secondary Outcomes; Figures 3 and 4; Supplementary Figure S1
RESULTS Results of syntheses	**20a**	For each synthesis, briefly summarise the characteristics and risk of bias among contributing studies.	pp. 6–9, Characteristics of Included Studies and Risk of Bias; pp. 9–13, outcome-specific results
RESULTS Results of syntheses	**20b**	Present results of all statistical syntheses conducted. If meta-analysis was done, present for each the summary estimate and its precision (e.g. confidence/credible interval) and measures of statistical heterogeneity. If comparing groups, describe the direction of the effect.	pp. 9–13, Primary and Secondary Outcomes; Figures 3 and 4; Supplementary Figure S1
RESULTS Results of syntheses	**20c**	Present results of all investigations of possible causes of heterogeneity among study results.	pp. 9–12, Galbraith plots (Figures 3C and 4C); pp. 14–15, Discussion
RESULTS Results of syntheses	**20d**	Present results of all sensitivity analyses conducted to assess the robustness of the synthesized results.	pp. 9–12, Leave-one-out sensitivity analyses (Figures 3B and 4B)
RESULTS Reporting biases	**21**	Present assessments of risk of bias due to missing results (arising from reporting biases) for each synthesis assessed.	pp. 4–5, Statistical Analysis (not formally assessed because <10 studies per outcome)
RESULTS Certainty of evidence	**22**	Present assessments of certainty (or confidence) in the body of evidence for each outcome assessed.	p. 9, Table 3 (GRADE evidence profile)
DISCUSSION	**23a**	Provide a general interpretation of the results in the context of other evidence.	pp. 14–15, Discussion
DISCUSSION	**23b**	Discuss any limitations of the evidence included in the review.	p. 15, Strengths and Limitations paragraph
DISCUSSION	**23c**	Discuss any limitations of the review processes used.	p. 15, Strengths and Limitations paragraph
DISCUSSION	**23d**	Discuss implications of the results for practice, policy, and future research.	pp. 15–16, Implications for Practice and Future Research; Conclusion
OTHER INFORMATION Registration and protocol	**24a**	Provide registration information for the review, including register name and registration number, or state that the review was not registered.	p. 3, Protocol Registration; p. 17, PROSPERO Registration (CRD420261478646)
OTHER INFORMATION Registration and protocol	**24b**	Indicate where the review protocol can be accessed, or state that a protocol was not prepared.	p. 3, Protocol Registration (PROSPERO record accessible using CRD420261478646)
OTHER INFORMATION	**24c**	Describe and explain any amendments to information provided at registration or in the protocol.	No amendments or deviations from the registered protocol.

Registration and protocol			
OTHER INFORMATION Support	**25**	Describe sources of financial or non-financial support for the review, and the role of the funders or sponsors in the review.	p. 17, Funding: None
OTHER INFORMATION Competing interests	**26**	Declare any competing interests of review authors.	p. 17, Conflicts of Interest: None
OTHER INFORMATION Availability of data, code and other materials	**27**	Report which of the following are publicly available and where they can be found: template data collection forms; data extracted from included studies; data used for all analyses; analytic code; any other materials used in the review.	p. 17, Data transparency

Table S2: Search Strategy.

Database	Search Terms	Search Field	Search Results
Pubmed	(“virtual reality” OR “smart glass*” OR “immersive” OR “non-immersive” OR “head-mounted display” OR “augmented reality” OR “mixed reality” OR “virtual therapy” OR “virtual environment” OR “virtual treatment” OR “visual distract*” OR “audiovisual distract*” OR “photic stimulation” OR “motion picture*” OR “watch* video*”) AND ("Prostate Biopsy" OR TRUS OR “transrectal prostate biopsy” OR “transrectal ultrasound guided prostate biopsy” OR “TRUS-PB”)	All Fields	49
Cochrane	(“virtual reality” OR “smart glass*” OR “immersive” OR “non-immersive” OR “head-mounted display” OR “augmented reality” OR “mixed reality” OR “virtual therapy” OR “virtual environment” OR “virtual treatment” OR “visual distract*” OR “audiovisual distract*” OR “photic stimulation” OR “motion picture*” OR “watch* video*”) AND ("Prostate Biopsy" OR TRUS OR “transrectal prostate biopsy” OR “transrectal ultrasound guided prostate biopsy” OR “TRUS-PB”)	All Text	28
Web of Science	(“virtual reality” OR “smart glass*” OR “immersive” OR “non-immersive” OR “head-mounted display” OR “augmented reality” OR “mixed reality” OR “virtual therapy” OR “virtual environment” OR “virtual treatment” OR “visual distract*” OR “audiovisual distract*” OR “photic stimulation” OR “motion picture*” OR “watch* video*”) AND ("Prostate Biopsy" OR TRUS OR “transrectal prostate biopsy” OR “transrectal ultrasound guided prostate biopsy” OR “TRUS-PB”)	All Fields	76
SCOPUS	TITLE-ABS-KEY (("virtual reality" OR "smart glass*" OR "immersive" OR "non-immersive" OR "head-mounted display" OR "augmented reality" OR "mixed reality" OR "virtual therapy" OR "virtual environment" OR "virtual treatment" OR "visual distract*" OR "audiovisual distract*" OR "photic stimulation" OR "motion picture*" OR "watch* video*") AND ("Prostate Biopsy" OR TRUS OR "transrectal prostate biopsy" OR "transrectal ultrasound guided prostate biopsy" OR "TRUS-PB"))	Title, Abstract, Keywords	101
EMBASE	(“virtual reality” OR “smart glass*” OR “immersive” OR “non-immersive” OR “head-mounted display” OR “augmented reality” OR “mixed reality” OR “virtual therapy” OR “virtual environment” OR “virtual treatment” OR “visual distract*” OR “audiovisual distract*” OR “photic stimulation” OR “motion picture*” OR “watch* video*”) AND ("Prostate Biopsy" OR TRUS OR “transrectal prostate biopsy” OR “transrectal ultrasound guided prostate biopsy” OR “TRUS-PB”)	All Fields	110

Table S3: Excluded records in full-text screening.

Title	Published year	Doi	Study	Exclusion Reason
Effects of virtual reality on perceived pain during transrectal biopsy	2021	10.1016/s2666-1683(21)00905-8	Deluca 2021	Conference abstract
Virtual reality immersion for reducing anxiety and pain during transperineal prostate biopsy: a prospective, randomized clinical trial	2023	10.1097/ju.0000000000003217.15	Kim 2023	Conference abstract
The effect of audio and audiovisual distraction on pain and anxiety in patients receiving outpatient perineal prostate biopsies: a prospective randomized controlled study	2025	10.3390/cancers17060959	Kaulfuss 2025	Wrong intervention
Effect of virtual reality (vr) as analgesia for trans-rectal ultrasound prostate biopsy (trus bx) on pain severity: a prospectively randomized study	2022	10.1111/bju.4_15743	Robinson 2022	Conference abstract
Is music or virtual reality (vr) interventions better for pain and anxiety control for patients undergoing transperineal (tp) prostate biopsy?	2026	10.1111/bju.70129	Chu 2026	Conference abstract
Use of virtual reality to reduce perioperative anxiety and pain in urologic interventions: a randomized controlled prospective study	2024	10.1016/s2666-1683(24)00059-4	Schreen 2024	Conference abstract
Virtual reality induced pain and anxiety relief in outpatient urological procedures: a post-marketing clinical investigation of realica	2025	-	Nct07338292	Study protocol
Effect of virtual reality goggles on anxiety, comfort and complications in patients undergoing transrectal ultrasound-guided prostate biopsy: a randomised controlled study	2025	-	Nct06959264	Study protocol
Feasibility of virtual reality assistance in prostate biopsy under local anesthesia	2024	-	Nct06274684	Wrong study design
The effect of virtual reality glasses application on pain, anxiety, and patient satisfaction during a transrectal prostate biopsy: a randomized controlled trial	2022	--	Nct05509114	Study protocol
Augmented reality-guided transperineal prostate biopsy: shifting the paradigm	2024	Https://dx.doi.org/10.5489/cuaj.8978	Cheng 2024	Wrong setting
The impact of virtual reality(vr)on patient experience undergoing prostate biopsy	2024	Https://dx.doi.org/10.1097/01.ju.0001009504.18450.e0.09	Moscardi 2024	Conference abstract
Virtual reality with biofeedback enhances tolerability of office based transperineal prostate biopsy	2024	Https://dx.doi.org/10.1097/01.ju.0001009504.18450.e0.10	Myrga 2024	Conference abstract
The effect of a virtual reality device (vrd, hypnovr) on pain, anxiety scores and satisfaction during transperineal targeted and systemic prostate biopsies	2023	-	Wen 2023	Conference abstract
Single-center, pilot study evaluating feasibility of virtual reality to alleviate pain during transperineal prostate biopsy	2023	Https://dx.doi.org/10.5489/cuaj.8579	Natesan 2023	Conference abstract
Impact of virtual reality on pain management in transrectal mri-guided prostate biopsy.	2023	10.3389/fpain.2023.1156463	Perenic 2023	Wrong study design
Assessment of a novel smartglass-based point-of-care fusion approach for mixed reality-assisted targeted prostate biopsy: a pilot proof-of-concept study.	2022	10.3389/fsurg.2022.892170	Sparwasser 2022	Wrong setting

Table S4: Detailed RoB 2 signaling-question responses and study-specific supporting justifications.

Study	Domain	Signaling-question assessment / supporting information	Judgment
Bozkul et al. 2026	D1 Randomization	1.1 Y; 1.2 Y; 1.3 N. Computer-generated block randomization was performed by an independent statistician, and allocation was concealed in opaque sealed envelopes. Baseline characteristics did not indicate a randomization problem.	Low
	D2 Deviations from intended interventions	2.1 Y; 2.2 Y; 2.3 N; 2.6 Y. Participants necessarily knew whether VR was being used, but no important intervention-related deviations were reported and groups received the intended care.	Low
	D3 Missing outcome data	3.1 Y. All 70 randomized participants were retained and analyzed; no VR-related withdrawal was reported.	Low
	D4 Measurement of outcome	4.1 N; 4.2 N; 4.3 Y; 4.4 Y; 4.5 PN. Anxiety was assessed with the same validated instrument in both groups. Participants knew their allocation and self-reported anxiety, so knowledge of intervention could potentially influence assessment, but there was no evidence that such influence was likely or differential.	Some concerns
	D5 Selection of reported result	5.1 PY; 5.2 N; 5.3 N. Trial registration was reported and the stated outcomes corresponded to those analyzed; no clear evidence of selective outcome or analysis choice was identified.	Low
Genç et al. 2022	D1 Randomization	1.1 Y; 1.2 PY; 1.3 N. Randomization was performed using Research Randomizer and the groups were comparable at baseline; allocation-concealment procedures were not described in detail.	Low
	D2 Deviations from intended interventions	2.1 Y; 2.2 Y; 2.3 N; 2.6 Y. VR could not be blinded, but no trial-related deviations from the assigned interventions were evident.	Low
	D3 Missing outcome data	3.1 Y. All 32 VR and 32 control participants contributing to the relevant comparison had outcome data reported.	Low
	D4 Measurement of outcome	4.1 N; 4.2 N; 4.3 Y; 4.4 Y; 4.5 PN. Pain was measured using the same VAS procedure in both groups. Participant awareness could potentially influence a subjective self-reported pain score, but there was no evidence that such influence was likely to differ systematically between groups.	Some concerns
	D5 Selection of reported result	5.1 NI; 5.2 PY; 5.3 PY. No accessible prospective analysis plan was identified, although the prespecified hypotheses and outcomes were described in the article; therefore some uncertainty regarding selective reporting remained.	Some concerns
Robinson et al. 2026	D1 Randomization	1.1 Y; 1.2 Y; 1.3 N. Block randomization with random block sizes was used and allocation was maintained in sealed envelopes; baseline characteristics were comparable.	Low
	D2 Deviations from intended interventions	2.1 Y; 2.2 Y; 2.3 N; 2.6 Y; 2.7 N. Participant/clinician blinding was not feasible, but the analysis was conducted by randomized assignment and investigators analyzing the data were blinded.	Low
	D3 Missing outcome data	3.1 N; 3.2 PY. Eighteen participants withdrew after randomization and there was some intervention crossover; nevertheless, the study used intention-to-treat analysis and retained most randomized participants.	Some concerns
	D4 Measurement of outcome	4.1 N; 4.2 N; 4.3 Y; 4.4 Y; 4.5 PN. Pain and anxiety were assessed using the same VAS-based methods in both groups. As subjective outcomes, they could potentially be influenced by participants' knowledge of receiving VR, but there was no evidence that such influence was likely to distort the measurements.	Some concerns
	D5 Selection of reported result	5.1 PY; 5.2 N; 5.3 N. The trial was prospectively registered and primary outcomes were clearly specified; no evidence of selective outcome or analysis reporting was identified.	Low
Toraman et al. 2024	D1 Randomization	1.1 Y; 1.2 NI; 1.3 N. Two-block randomization using random.org was reported, but concealment of the allocation sequence before assignment was not clearly described.	Some concerns
	D2 Deviations from intended interventions	2.1 Y; 2.2 Y; 2.3 N; 2.6 Y. Participants and personnel were aware of the intervention, but there was no evidence of deviations arising from the trial context.	Low
	D3 Missing outcome data	3.1 Y. No participants were lost to follow-up; 35 participants per group were analyzed.	Low
	D4 Measurement of outcome	4.1 N; 4.2 N; 4.3 Y; 4.4 Y; 4.5 PN. VAS and STAI-I were administered identically to both groups. Participant awareness could potentially influence these subjective self-reported outcomes, but there was no evidence that it likely affected assessment differentially.	Some concerns

	D5 Selection of reported result	5.1 PY; 5.2 N; 5.3 N. Prespecified outcomes and analyses were described, with no clear evidence of selective reporting.	Low
